# Ultrasound-targeted microbubble destruction for chemotherapeutic drug delivery to solid tumors

**DOI:** 10.1186/2050-5736-1-10

**Published:** 2013-07-01

**Authors:** Hong Chen, Joo Ha Hwang

**Affiliations:** 1 Division of Gastroenterology, Department of Medicine, University of Washington, 1959 NE Pacific St, Seattle, WA 98195, USA

**Keywords:** Ultrasound, Microbubbles, Ultrasound contrast agent, Ultrasound-targeted microbubble destruction, Cancer, Targeted drug delivery

## Abstract

Ultrasound-targeted microbubble destruction (UTMD) is a promising technique for non-invasive, targeted drug delivery, and its applications in chemotherapeutic drug delivery to solid tumors have attracted growing interest. Ultrasound, which has been conventionally used for diagnostic imaging, has evolved as a promising tool for therapeutic applications mainly because of its ability to be focused deep inside the human body, providing a modality for targeted delivery. Although originally being introduced into clinics as ultrasound contrast agents, microbubbles (MBs) have been developed as a diagnostic and therapeutic agent that can both be tracked through non-invasive imaging and deliver therapeutic agents selectively at ultrasound-targeted locations. Whereas free drugs often possess harmful side effects, their encapsulation in MBs and subsequent local release at the targeted tissue by ultrasound triggering may help improve the margin of safety. In the past 10 years, the feasibility and safety of UTMD have been extensively tested using normal animal models. Most recently, a growing number of preclinical studies have been reported on the therapeutic benefits of UTMD in the delivery of chemotherapeutic drugs to various malignant tumors, such as brain, liver, eyelid, pancreas, and breast tumors. Increased drug concentration in tumors and reduced tumor sizes were achieved in those tumors treated with UTMD in combination with chemotherapeutic drugs, when compared to tumors treated with chemotherapy drugs alone. This review presents an overview of current preclinical applications of UTMD in chemotherapeutic drug delivery for the treatment of cancers along with a discussion of its future developments.

## Introduction

Cancer is a leading cause of death worldwide. Systemic chemotherapy is the main treatment available for a wide variety of cancers; however, the lack of tumor response to chemotherapy is well documented in many cancer types [1]. The main hindrance for the distribution of anticancer agents to the tumor site is the poorly organized tumor vasculature, irregular blood flow, and high interstitial pressure within the tumor tissue [2-4]. This insufficient response of cancer to chemotherapeutic drugs have urged the need for developing new strategies for enhancing localized drug delivery to tumors while minimizing systemic side effects. Ultrasound (US) in combination with microbubbles (MBs) has been demonstrated as a new promising strategy for targeted drug delivery to tumors [5,6]. US is the most widely used diagnostic medical imaging modality, and its potential in therapeutics has been explored for several decades [7,8]. It is non-invasive, has no hazardous ionizing radiation, and can transmit energy deep into the human body. Moreover, the ultrasound beam can be focused to a small focal region (usually in the order of millimeters) deep into tissues, which allows highly precise targeting of diseased tissues during therapy. This relatively safe and non-invasive transmission of energy deep into the body is the key to ultrasound-mediated drug delivery [9].

MBs, bubbles with diameters of less than 10 μm, are made of a phospholipid, surfactant, albumin, or synthetic polymer shell filled with high molecular weight gas with a very low water solubility (e.g., sulfurhexafluoride or perfluorepropane gas) [10]. Bioactive substances (e.g., genes, drugs, proteins, and gene silencing constructs) can be attached to or incorporated in the MBs. Although originally being introduced into clinics as US contrast agents for enhancing the ultrasound signals from the blood pool, MBs have been developed as an agent with both diagnostic and therapeutic capabilities.

The technique that uses low-intensity US in combination with MBs is often called US-targeted MB destruction (UTMD) [11-13]. Compared to conventional systemic delivery of chemotherapy drugs, drug delivery by UTMD is a targeted delivery strategy. Targeted drug release can be achieved when an external US beam is focused within a tumor, so only MBs passing through the beam interact with the US energy. While many externally triggered drug release techniques have been explored, UTMD is unique, in that it can facilitate the drug to overcome the tumor barriers to reach tumor cells. When MBs are exposed to an US field, the US mechanical wave causes them to cavitate, which is a broad term for US-induced activities of bubbles, including their formation, oscillation, and collapse. The interaction of the US-activated MBs with tissue results in thermal and mechanical effects. For drug delivery applications, the US parameters were selected to induce mechanical effects while reducing the chance of generating heat as it can lead to thermal ablation of the tumor [14,15]. These mechanical effects have the potential to increase microvessel permeability of drugs, enhance drug penetration through the interstitial space, and increase tumor cell uptake of the drugs [9,16,17], thus enhancing the antitumor effectiveness of the drugs.

The topic of US-medicated drug delivery has been discussed in a number of excellent reviews [9,16,18-21]. The purpose of this article is to review the progress in therapeutic benefit studies using UTMD to enhance the delivery of chemotherapy drugs to solid tumors, as it is more closely related to future clinical applications than other *in vivo* studies using UTMD in the delivery of model drugs to normal animal models.

### Current UTMD technique for tumor drug delivery

The UTMD technique uses US to force dynamic behaviors of MBs to increase tumor uptake of chemotherapeutic agents at targeted locations. The use of xenograft or orthotopic tumor models in evaluating the therapeutic benefits of UTMD has been reported in the literature. The US transducer is placed extracorporeally and directed toward deeply located tumor sites. High spatial resolution can be achieved using focused US with a beam-width on the order of millimeters. Coupling between the transducer and the animal skin is achieved through US gel or water. The therapeutic drugs can be co-administrated with MBs through intravenous injection, taking advantage of the enhanced delivery effect of UTMD, or carried by the MBs on their shell or core in various different ways, taking advantage of both increased delivery and increased local concentrations of the delivered substance [11]. After bolus injection, MBs arrive at the tumor site in approximately a few seconds, and their lifetime is approximately 5 min [22]. For continuous injection, MBs can be effective for longer time. After MBs reached the targeted tumor region, US is then focused on the tumor in an effort to locally deliver the drug at the diseased site as the interaction of the MBs and the tissue only happens at the targeted location. To treat a larger area, multiple spots are treated or the focus of the transducer is scanned within the tumor. Although intravenous injection was used most commonly, invasive direct intratumorally injection of MBs was used by two groups [23,24]. Currently, US treatment is normally combined with magnetic resonance imaging (MRI) or US imaging, assisting the treatment with planning, monitoring, and evaluation strategies [25,26].

Same as other therapeutic applications of US, UTMD is a double-edged sword, in that on the one hand it is requisite in promoting the transport of drugs into cells and on the other hand it may damage tissues. The key to achieve the desired bioeffects for efficient material transfer is complimentary adjustments in US and MB parameters. One of the most commonly used strategies is to find a combination of parameters that induce bubble activity sufficient to allow drug extravasation from capillary walls without irreversibly damaging the endothelial cells [27]. MB parameters include their sizes, shell compositions, doses, and injection sites. The common choice of the US center frequency is low megaHertz (e.g., 1–5 MHz) to be close to the resonant frequencies of MBs. The peak rarefactional pressures are normally low (e.g., approximately 0.5 MPa) to induce cavitation while minimizing the potential for tissue damage. Pulsed waves are most commonly used with pulse durations on the order of 10 ms. The pulse repetition frequency (PRF) is normally close to the heart rate, e.g., 1–10 Hz, to allow sufficient time between pulses for MBs to re-enter the vasculature. In some studies, continuous waves were used with the US on for several seconds followed by several seconds off, while the intensities were so low that no thermal effect was induced [28,29]. Although successful treatment of tumors was demonstrated in those studies, continuous waves are not optimal for drug delivery application in consideration of the short lifetime of MBs upon sonication.

In the last 10 years, extensive feasibility studies were performed using UTMD in the delivery of model drugs to normal animal models [30,31]. Most recently, a growing number of preclinical studies have been reported on the efficacy of UTMD in treating tumors with chemotherapeutic drugs. Brain and liver cancers have been the most broadly explored diseases for the application of UTMD; there have also been publications on other cancers, such as eyelid, pancreas, and breast cancers. The amount of drug delivered to the tumor was commonly quantified by high-performance liquid chromatography or using a spectrophotometer, and the tumor volumes were quantified based on direct caliber measurements or images obtained with MRI, US, CT, or biophotonics. These efficacy studies have shown promising results: increased drug concentration in tumors and reduced tumor sizes were achieved in tumors treated with UTMD in combination with chemotherapeutic agents, in comparison to tumors treated using conventional systemic chemotherapy.

## Preclinical applications of UTMD in oncology

### UTMD therapy for brain cancer

Current methods of chemotherapeutic agent delivery have limited success for patients with glioma tumors because of the existence of the blood–brain barrier (BBB) in the brain. Although brain tumor capillaries may have increased permeability, the BBB remains a formidable obstacle in the treatment of brain malignancies. UTMD has been established as a promising technology for non-invasive, transient, reversible, and local BBB disruption [32,33].

The first efficacy study using UTMD for chemotherapy delivery to animals with glioma tumors was published by Liu et al. [34]. The rat brain glioma model was developed by direct intracranial injection of gliomal tumor cells. Following intravenous injection of MBs (SonoVue®, Bracco Diagnostics, Milan, Italy), 1,3-*bis*-(2-chloroethyl)-1-nitrosourea (BCNU), a common chemotherapeutic agent for brain tumors, was intravenously injected. Then the targeted tumor region was sonicated transcranially using a MR-monitored focused US transducer with 0.4-MHz frequency, 0.62-MPa pressure, 10-ms pulse length, 1-Hz PRF, and 30-s sonication time (Table 1). These parameters are within the range of most commonly used parameters for BBB opening. After US treatment, it was found that BCNU concentration increased by nearly two times at the UTMD-treated brain compared with untreated control. The median survival time for the control, BCNU, and BCNU + UTMD were 28.5, 33 (p=0.023 relative to control), and 53 (p=0.0015 relative to control) days, respectively. This study demonstrated the feasibility of UTMD for increasing localized chemotherapeutic drug delivery with chemotherapeutic doses in the tumor region to suppress tumor growth and prolong animal survival. It is unclear why the animal survival declined rapidly after 50 days. It is important to investigate the systemic toxicity of the treatment and biodistribution of the chemotherapeutic agent to demonstrate conclusively the safety and efficacy of this approach.

**Table 1:** Summary of UTMD in brain tumor therapy studies

**Reference**	**Animal model**	**Chemo drug**		**MB**		**US**					
		**Type**	**Dose**	**Type**	**Dose**	**Frequency (MHz)**	**Pressure (MPa)**	**Pulse length (ms)**	**PRF (Hz)**	**Exposure time of each treatment (s)**	**Treatment days**
Liu et al. [34]	Rat xenograft brain glioma	BCNU	13.5 mg/kg	SonoVue®	2.5 μg/kg	0.4	0.62	10	1	30	1
Ting et al. [35]	Rat xenograft brain glioma	BCNU	-	BCNU-loaded MBs	-	1	0.7	10	5	60 s per sonication and repeated twice	2
Treat et al. [36]	Rat xenograft brain glioma	Dox	5.67 mg/kg	Definity®	0.01-0.02 ml/kg	1.7	1.2	10	1	60–120 s per sonication and repeated to cover the tumor	1

Recently, multifunctional MBs capable of BCNU loading, BBB opening induction, and local BCNU release were developed and used in UTMD-enhanced drug delivery to brain tumors [35]. The US treatment parameters were similar to the above study (Table 1). However, instead of single transcranial treatment, the study was performed multiple times with a craniotomy. Treatments were on two consecutive days, and each day, two repeated treatments were performed. Encapsulation of the BCNU in MBs (BCNU-MBs) prolonged BCNU's circulatory half-life significantly and reduced the systemic toxicity. The median survival for the untreated control, BCNU, and BCNU-MBs + US was 29, 29.5, and 32.5 days, respectively. The maximum survival for the three groups was 38, 43, and 59 days, respectively. The increase in the maximum survival was significant comparing the US-treated group and the control. However, it is not clear from this paper whether the increase in median survival is significant. This study demonstrated that the use of MBs carrying chemotherapeutic agents in combination with US exposure is a feasible approach to transport chemotherapeutic drugs across the BBB and improve therapeutic outcome. Although the ultrasound parameters used in this study had been optimized based on studies using normal animal model, the median survival time of 32.5 days achieved with BCNU-MBs + US was much lower than the 53 days reported in the above study (Liu et al. [34]) using BCNU + MBs + US while the median survival time of their control groups was close (28.5 and 29 days, respectively). Future work is needed to compare these two strategies to demonstrate whether BCNU-loaded MBs can further improve the therapeutic efficacy.

Doxorubicin (Dox) is another chemotherapeutic agent with demonstrated antineoplastic efficacy and widespread clinical use but cannot cross the intact BBB. The first study that demonstrated the therapeutic benefit of delivering Dox across the BBB using UTMD was published by Treat et al. [36] (Table 1). Rats with implanted brain glioma tumors were treated transcranially with MRI-guided focused ultrasound. Sonication after bolus injection of MBs (Definity®, Lantheus Medical Imaging, North Billerica, MA, USA) was repeated every 5 min in overlapping square pattern until the entire projected area of the tumor on the MRI image had been exposed to the acoustic focus. For each sonication, Dox hydrochloride encapsulated in long-circulating pegylated liposomes was injected immediately after the administration of MBs. Delayed tumor growth was demonstrated by the increase of tumor doubling time constants from 2.3 days for the untreated control to 3.7 days for UTMD + Dox. The median survival times for the control, US only, Dox only, and UTMD + Dox were 25, 25, 29, and 31 days, respectively. The median survival time of the last group was significantly higher than that of the control (*p* = 0.0007). The demonstration of increased antitumor efficacy of Dox by UTMD represents a major milestone in the development of this technique for clinical application. However, the increase in survival time is modest, suggesting that future optimization is needed to further improve the efficacy.

### UTMD therapy of liver cancer

Hepatocellular carcinoma ranks as the third most common cause of death from cancer worldwide. Unfortunately, liver cancer resists most chemotherapy drugs. Chemotherapy drugs are effective in only a small proportion of liver tumors, and the responses often are not durable. UTMD has been proposed as a new strategy for chemotherapy drug delivery to liver tumors.

Kang et al. [29] investigated the possibility of using UTMD with docetaxel-loaded lipid MBs (Dox-MB) to inhibit the growth of liver tumors in an orthotpic animal model by releasing the drug locally and enhancing its delivery to carcinoma tissues. Instead of using a focused ultrasound transducer as used in most studies, a 0.3-MHz non-focused US transducer was used for the treatment with an intensity of 2 W/cm^2^. Tumors were exposed for 10 s followed by 10 s off, with a total treatment duration of 6 min (Table 2). The treatments were performed on three separate times on days 1, 4, and 7. It was found that UTMD with Dox-MBs inhibited the growth of the liver tumors by decreasing proliferation and promoting apoptosis. Compared with the untreated control, the mean survival time of the Dox-MBs + US group increased from 23.6 to 36.8 days (*p* < 0.01) and the extensive metastasis rate decreased from 100% to 0% (*p* < 0.01). The efficacy of UTMD in this study can be further improved, as the ultrasound frequency was much lower than the resonant frequency of the MBs. Moreover, as mentioned before, the strategy of turning ultrasound on for 10 s followed by 10 s off was not optimized in considering MB dynamics.

**Table 2:** Summary of UTMD in liver tumor therapy studies

**Reference**	**Animal model**	**Chemo drug**		**MB**		**US**					
		**Type**	**Dose**	**Type**	**Dose**	**Frequency (MHz)**	**Pressure (MPa)**	**Pulse length (ms)**	**PRF (Hz)**	**Exposure time of each treatment (s)**	**Treatment days**
Kang et al. [29]	Rabbit orthotopic liver tumor	Dox	2 mg/rat	Dox-loaded lipid MBs	4.4-6.4 × 10^9^ MBs/rabbit	0.3	Intensity: 2 W/cm^2^	10 s on	-	360	3
								10 s off			
Li et al. [28]	Mice xenograft liver tumor	HCPT	4 mg/kg	HCPT-loaded lipid MBs	1.1 × 10^9^ MBs/mice	1	Intensity: 2 W/cm^2^	10 s on	-	3,600	7
								10 s off			
Cochran et al. [37]	Rat xenograft liver tumor	Dox	167 μg/rat	Dox-loaded polymer MBs	-	12–5	MI: 0.40–0.45	Doppler mode	1,000	1,200	1

One of the obstacles for clinical use of UTMD is that MBs have limited loading capacity of chemotherapy drugs because they have a thin shell (few nanometers) and gas core. Instead of working to increase the loading of the MBs, Li et al. [28] used a powerful antitumor drug: 10-hydroxycamptothecin (HCPT). The required dose of HCPT for cancer therapy is over 20 times lower than that of paclitaxel or doxorubicin. Therefore, a smaller dose of drug is required for therapeutic efficacy. HCPT-loaded MBs (HCPT-MBs) were prepared and injected intravenously, followed by sonication. Focused US parameters were the same as the above study with the exception that the frequency was 1 MHz instead of 0.3 MHz and treatment was performed once a day for seven consecutive days (Table 2). The group treated with HCPT-MBs + US exhibited a fivefold higher HCPT concentration in tumor tissues at 4 h post-administration of HCPT and a 1.5-fold higher tumor inhibition rate compared to that of HCPT-MBs alone at the second day after the last treatment. The results obtained in this study indicate that HCPT-MBs may be capable of overcoming the current limitation of drug payload of MBs depending on the agent used. Long-term survival studies need to be performed to determine whether the tumor inhibition effect can be sustained.

Doxorubicin-loaded polymer MB is another platform proposed for UTMD. Using a rat liver cancer model, Cochran et al. [37] examined the biodistribution and delivery of polymer MB loaded with ^14^C-labeled doxorubicin. The tumor was continuously insonated using a diagnostic ultrasound scanner (Philips HDI-500, Philips, Bothell, WA, USA) with a linear 12–5-MHz transducer. The scanner was operated in Doppler mode with a mechanical index of 0.4–0.45 and a PRF of 1 kHz (Table 2). Compared to the group treated by free doxorubicin, animals treated with Dox-MBs + US had significantly lower plasma Dox concentration, lower drug levels in the myocardium, higher drug levels in tumors, and less tumor growth. Autoradiography of tumor sections showed that the majority of Dox was restricted to the periphery of the tumor. It is hypothesized by the authors that after the drug-loaded MBs were fragmented by US to nanoparticles at the tumor site, they are capable of escaping the tumor vasculature, accumulating within the tumor, and slowly releasing the drug. This study demonstrated that Dox-loaded polymer MBs is a promising platform for chemotherapy drug delivery. It also demonstrated that a commercial ultrasound scanner can be successfully used for drug delivery with UTMD, which may lead to easier clinical translation of this technique. However, future work is needed to develop a more sophisticated ultrasound regimen to enhance the penetration of drugs into tumors.

### UTMD therapy of other cancers

Besides brain and liver cancers, UTMD has also been explored in the treatment of other cancers. Examples of its application in eyelid, pancreas, and breast cancers are described below.

Melanomas of the eye can involve various ocular structures, including the eyelid, conjunctiva, and uvea, but their treatment is difficult. UTMD has been used in the treatment of mice xenograft eyelid tumors [23]. An antitumor drug, bleomycin, was mixed with Optison® MBs (Amersham Health, Princeton, NJ, USA) and then injected directly into the center of the tumor using a syringe. Immediately after the injection, a Sonitron 2000^TM^ probe (Rich Mar Inc., Inola, OK, USA), a non-focused transducer designed for sonoporation, was placed directly on the tumor surface for US sonication with the following parameters: 1-MHz frequency, 2-W/cm^2^ power density, 50% duty cycle, and 240-s duration (Table 3). The antitumor effect of bleomycin was observed when the treatment was used in conjunction with UTMD. Immunostaining revealed that a high rate of bleomycin was introduced into the tumor cells. No abnormalities such as inflammation or degeneration in any tissues, such as brain, lung, liver, or heart, were observed on day 14. This study demonstrated that UTMD is a promising technology; however, the direct intratumoral injection is an invasive approach, and whether it can increase drug delivery when compared with intravenous injection is not clear.

**Table 3:** Summary of UTMD in other tumor therapy studies

**Reference**	**Animal model**	**Chemo drug**		**MB**		**US**					
		**Type**	**Dose**	**Type**	**Dose**	**Frequency (MHz)**	**Pressure (MPa)**	**Pulse length (ms)**	**PRF (Hz)**	**Exposure time of each treatment (s)**	**Treatment days**
Sonoda et al. [23]	Mice xenograft eyelid tumor	Bleomycin	0.003–0.025 mg/mouse	Optison®	10 μL/mouse	1	Intensity: 1 W/cm^2^	-	-	240	4
Tinkov et al. [38]	Rat orthotopic pancreas tumor	Dox	140 μg/rat	Dox-loaded lipid MBs	3.14 × 10^9^ MB/rat	1.3	1.2	-	-	1,200	2
Sorace et al. [39]	Mice xenograft breast tumor	Taxol	0.22 mg/mouse	Definity®	30 μL/mouse	1	0.1–2.0	1,000	5	300	6

Pancreatic ductal adenocarcinoma (PDA) remains one of the most difficult cancers to treat, with a 5-year relative survival of 6%. In a study by Tinkov et al. [38], PDA cells were orthotopically grown in the pancreas tails of nu/nu mice. Following the intravenous injection of Dox-loaded MBs, a diagnostic ultrasound system, Sonos 5500 (Philips, Eindhoven, The Netherlands), operating in ultraharmonic mode was used for the sonication. A burst of four frames of US every four cardiac cycles was delivered during a constant rate infusion of MBs for 20 min (Table 3). A 12-fold increase in tissue concentration was achieved in targeted tumors with a threefold decrease in tumor growth compared to the contralateral control tumor.

Breast cancer ranks second as a cause of cancer death in women (after lung cancer). Chemotherapy may be used before surgery removal to shrink the cancer or after the surgery to get rid of any cancer cells that may be left behind after surgery or to reduce the risk of the cancer coming back. Sorace et al. [39], using a subcutaneous breast cancer animal model, explored the effect of mechanical index (MI) on the *in vivo* tumor growth and necrosis (Table 3). MI is defined by the peak negative pressure of the ultrasound wave divided by the square root of its center frequency. MB-mediated focused US therapy using a MI value of 0.5 resulted in the highest impediment in tumor growth over the 3-week treatment period and also the highest degree of necrosis per tumor volume, followed by therapy using MI of 1.0, 0.1, and 2. A MI of 2.0 exhibited very little tumor necrosis, 17 times less than that with a MI of 0.5. Excessively high MI values have been shown to cause bursting of capillaries [40], which in turn could decrease drug delivery to the tumor cells. Conversely, when a low MI is used, there is thought to be little MB cavitation, creating no enhanced drug delivery. All the previous mentioned studies were performed with one fixed group of parameters. This is the first study that explored the effect of acoustic parameters on the efficacy of UTMD cancer treatment.

## Current limitations and future perspectives

The preclinical data appear promising to date for UTMD-mediated delivery of chemotherapy agents into tumors. However, there are still a number of concerns and obstacles that have slowed its progress toward clinical translation.

As pointed out earlier, although MBs are relatively large agents, the amount of drug that can be attached to the bubble surface or incorporated into the internal lipid layer is limited because of its thin shell and gas core. Using an antitumor drug with powerful activity is a promising approach; however, this drug may not be applicable to all types of tumors. The loading capacity can be increased if multilayers or additional oil layers are used. However, such MBs may significantly change their size and acoustic behavior. Insufficient loading capacities can be circumvented by co-administration of the therapeutic substance with MBs. With this method, no limitations exist for the amount of bioactive drugs. However, this strategy can only take advantage of transiently increased local permeability and not of increased local concentrations of the delivered drugs. In the future, new drug-specific MBs that can load optimal amounts of the drug without losing their acoustic behavior need to be developed.

Although the circulation time of MBs has increased in the past several years by improving their manufacturing techniques, this remains to be a concern for UTMD drug delivery. MBs are typically administered into a peripheral vein, so only a small amount of agent will pass through a tumor in a given circulatory cycle. Multiple circulations are necessary to allow destruction of enough agents to increase local concentration significantly. Therefore, the circulation time needs to be sufficient enough to allow higher amount of drug to be delivered to the targeted region. MB circulation times increase significantly with MB size, which is expected given the relationship of bubble dissolution to their diameter [41]. Using monodispersed MBs with larger sizes may improve the efficacy of UTMD treatment [42] not only because larger bubbles may generate larger mechanical effect on the tissue but also because they have longer circulation times.

Several different US systems have been used for UTMD, including focused ultrasound transducers, non-focused ultrasound transducers, and diagnostic US systems. Focused transducers have the advantage of high spatial resolution for tumor treatment as the beam-width can be focused to a few millimeters; however, to treat larger tumor areas, scanning will be needed, which requires longer treatment times. The non-focused transducers have the advantage of covering larger tumor area; however, the downside is the potential to affect non-tumor tissue along the acoustic path. The ability to use diagnostic US systems for therapeutic application is attractive, as it will simplify the clinical translation of the UTMD technique. However, the frequency of the imaging transducers and the pulse sequences are not necessarily optimized for drug delivery. Optimal US modalities have to be designed to improve its efficiency in MB activation.

A major challenge in the future clinical application of UTMD is the control over the procedure to ensure both safe and effective treatment. Extensive studies have demonstrated that cavitation is the main mechanism for UTMD-enhanced drug delivery [6,43]. Cavitation detection techniques have been developed to monitor the acoustic emissions from the oscillating MBs during sonication [44-46]. The strength of these emissions has been shown to be correlated with the extravasation of delivered agents [25]. The emissions have also been used as the basis for a feedback control algorithm to actively control US exposures to ensure safe treatment [47]. However, these studies were performed using non-tumor-bearing animals, while no investigation of cavitation activity was reported in all the previously mentioned studies. In the future, cavitation monitoring techniques should be incorporated to assist treatment outcome prediction and ensure safe treatments.

For future clinical translation, animal models that better recapitulate human tumors are needed. For example, it is known that xenograft and orthotropic pancreatic cancer models lack the prominent stromal matrix separating blood vessels from tumor cells, observed in human tumors. The stromal matrix has been demonstrated to be an important barrier to drug delivery in pancreatic cancer [48]. Thus, the success achieved in drug delivery using xenograft and orthotropic pancreatic cancer models may not be directly translatable to clinical application. Meanwhile, larger animal models are needed to scale up the treatment. Most recently, studies of UTMD-enhanced model drug delivery to normal monkey brain were reported [49,50]. Although no therapeutic agents were used, these preliminary results demonstrate the feasibility of performing the treatment on large animals more similar in scale to humans. Future successful preclinical applications of UTMD in realistic tumor models that are similar to human tumors should pave the way for clinical applications.

Last but not least, further evaluation of the safety of UTMD-mediated chemotherapy delivery is needed. There appears to be a consensus that a MI value of 0.4 represents the threshold for MB-induced bioeffects. Bioeffects, such as microvascular leakage, petechiae, cardiomyocyte death, inflammatory cell infiltration, and premature ventricular contractions, have been observed in animals *in vivo*[51]. For diagnostic applications, these bioeffects raise safety concerns; however, for therapeutic applications, MBs are used to induce bioeffects for therapeutic benefits, for example, to enhance vascular permeability of chemotherapy drugs. Assessment of safety for therapeutic applications must take a different form than for diagnostic applications [52]. Currently, the safety of UTMD in cancer therapy is mainly evaluated by animal survival. The systemic toxicity of the treatment needs to be investigated to demonstrate conclusively the safety of this technique.

## Summary

The use of UTMD as a tool for drug delivery has an enormous clinical potential in oncology. Whereas free drugs often possess harmful side effects, their encapsulation in MBs and subsequent local release and uptake in the target tissue by US triggering may help to improve the therapeutic outcome. A growing number of preclinical studies have successfully demonstrated the therapeutic benefits of UTMD in the delivery of chemotherapeutic drugs to various malignant tumors, such as brain, liver, eyelid, pancreas, and breast tumors. However, future improvements are needed in (1) the manufacture of MBs with longer circulation times and higher loading capacities without significantly altering their acoustic behavior, (2) the optimization of US modalities to improve their efficiency in MB activation, (3) the incorporation of cavitation monitoring techniques to assist in treatment monitoring, and (4) the evaluation of therapeutic benefits in realistic tumor models that are similar to human tumors. Nevertheless, the successful results already obtained in preclinical applications give great hope to future clinical translation of UTMD in cancer treatment.

## Competing interests

The authors declare that they have no competing interests.

## Authors’ contributions

HC wrote the draft of the manuscript. JH revised it critically and gave final approval of the version to be published. Both authors read and approved the final manuscript.
